# The transcription factor complex LMO2/TAL1 regulates branching and endothelial cell migration in sprouting angiogenesis

**DOI:** 10.1038/s41598-022-11297-3

**Published:** 2022-05-04

**Authors:** Yoshihiro Yamada, Yi Zhong, Shiho Miki, Akiko Taura, Terence H. Rabbitts

**Affiliations:** 1 The Central Biomedical Laboratory, Aino University School of Health Science, 4-5-11 Higashi-ohta, Ibaraki, Osaka 567-0012 Japan; 2 David Rubenstein Pancreatic Cancer Research Center, Memorial Sloan-Kettering Cancer Center, 417 East 68th Street, New York, NY 10065 USA; 3 Institute of Cancer Research, 15 Cotswold Road, Sutton, London, SM2 5NG UK

**Keywords:** Developmental biology, Molecular biology, Molecular medicine, Pathogenesis

## Abstract

The transcription factor complex, consisting of LMO2, TAL1 or LYL1, and GATA2, plays an important role in capillary sprouting by regulating VEGFR2, DLL4, and angiopoietin 2 in tip cells. Overexpression of the basic helix-loop-helix transcription factor LYL1 in transgenic mice results in shortened tails. This phenotype is associated with vessel hyperbranching and a relative paucity of straight vessels due to DLL4 downregulation in tip cells by forming aberrant complex consisting of LMO2 and LYL1. Knockdown of LMO2 or TAL1 inhibits capillary sprouting in spheroid-based angiogenesis assays, which is associated with decreased angiopoietin 2 secretion. In the same assay using mixed TAL1- and LYL1-expressing endothelial cells, TAL1 was found to be primarily located in tip cells, while LYL1-expressing cells tended to occupy the stalk position in sprouts by upregulating VEGFR1 than TAL1. Thus, the interaction between LMO2 and TAL1 in tip cells plays a key role in angiogenic switch of sprouting angiogenesis.

## Introduction

Sprouting angiogenesis is the main form of angiogenesis in which tip cells migrate into avascular areas, along with proliferation of stalk cells, to form new tubes^1^, and is stimulated by soluble chemoattractants and mitogens produced in areas with insufficient oxygen and nutrients. It is a two-step process initiated in quiescent endothelial cells: firstly, they have to be liberated through mural cell detachment and basement membrane degradation, and secondly, the cells are activated and give rise to tip cells, which inhibit the process in neighboring endothelial cells. This specification of endothelial cells into tip vs. stalk cells occurs via lateral inhibition by Delta-like ligand 4 (DLL4)/Notch signaling^2,3^. During development, DLL4 regulates vessel branching by inhibiting endothelial tip cell formation. Heterozygous deletion of DLL4 in mice results in a hyperbranching blood vessel phenotype. Vascular endothelial growth factor (VEGF)-A is a major proangiogenic chemoattractant and mitogen that is secreted from hypoxic sites and that binds to VEGF receptor 2 (VEGFR2) on the surface of tip cells. VEGF-A/VEGFR2 signaling induces formation of filopodia and enhances DLL4 expression in tip cells. DLL4-mediated activation of Notch in neighboring endothelial cells inhibits the tip cell phenotype and promotes differentiation into stalk cells with higher proliferative activity and fewer filopodia. In stalk cells, the expression of VEGFR2 is downregulated while that of VEGFR1 (a scavenger receptor of VEGF) is activated. Following anastomosis of new blood vessels and an increase in oxygen, activated endothelia revert to a quiescent state. This multistep process of sprouting angiogenesis is tightly regulated and only achieved via finely tuned transcriptional activation and inactivation^4^.

Several transcription factors that play important roles in angiogenesis have been identified. Among these, the LIM-only protein LMO2 is necessary for embryonic angiogenesis. A highly disorganized vascular system was observed in LMO2^−/−^ chimeric mice, which indicates an essential regulatory role for LMO2 in angiogenesis^5^. When LMO2^−/−^ embryonic stem cells were subcutaneously implanted into nude mice, a complete defect in vascular sprouting and vascular tree formation was observed during development of teratocarcinoma^6^, which indicates a key role for LMO2 in sprouting angiogenesis and vascular tree formation. Similarly, both TAL1 and GATA2 are needed for embryonic angiogenesis^7–10^ and tube formation^11,12^. LYL1 is a basic helix-loop-helix domain containing a transcription factor that is structurally related to TAL1^13^. LYL1 can bind to LMO2 and is therefore an alternative member of the LMO2 transcription factor complex. While TAL1 is exclusively expressed in angiogenic (activated) endothelial cells, LYL1 is also expressed in quiescent endothelium^14^. In LYL1^−/−^ mice, which exhibit relatively minor vascular system anomalies compared with TAL1 knockout mice, vascular sprouting is enhanced, resulting in increased neovasculature in tumor xenografts. Reduced vascular stabilization via LYL1 transcriptional activation results in increased vascular permeability and a larger vascular lumen^14,15^. In contrast, TAL1 plays a variety of roles in embryonic cardiovascular development and its expression is upregulated during angiogenesis^16–18^. Considering the vascular stabilizing effect of LYL1 in the quiescent endothelium, and the positive roles of TAL1 in the angiogenic endothelium, changing the LMO2 binding partner from LYL1 to TAL1 in the endothelium could trigger initiation of vascular sprouting.

LMO2 can directly bind to TAL1 or LYL1 and GATA2 to form a multimeric protein complex via its LIM domain zinc-finger-like structure^19–26^. This protein complex functions as a transcription factor (hereafter referred to as the LMO2 transcription factor complex) and regulates the expression of hundreds of hematopoiesis- and angiogenesis-related downstream genes^27^. Among angiogenesis-related genes, VE-cadherin, angiopoietin 2, VEGFR2, the VEGF co-receptor neuropilin 2, and DLL4 have been identified as direct targets of the LMO2 transcription factor complex^11,28–30^. VE-cadherin is a key component of endothelial adherens junctions and plays an important role in regulating vascular integrity and permeability. VEGF promotes rapid endocytosis of VE-cadherin, and Notch/VEGFR-regulated differential dynamics of VE-cadherin junctions drive competitive endothelial tip cell rearrangement during sprouting angiogenesis^31,32^. VEGFR2-DLL4-Notch signaling constitutes a negative feedback loop that is essential for tip and stalk cell selection. Angiopoietin 2 secreted from tip cells is thought to be another soluble factor important for endothelial cell activation^33^. It also binds to integrin, which activates Rac1 signaling and promotes endothelial cell migration^34^.

In the adult body, in which blood and lymphatic circulation is at a steady state, blood vessel endothelia are mainly quiescent and neovascularization is largely inhibited. In hypoxia, tissue damage, inflammation, and tumor growth, chemical signals produced from those sites activate endothelial cells in existing blood vessels. To investigate these dynamic transcriptional processes during sprouting angiogenesis, with a focus on the quiescent/angiogenic switch in the endothelium and competition between tip cells in sprouts mediated by the LMO2 transcription factor complex, we analyzed LYL1-overexpressing transgenic mice and discovered that their short and deformed tail phenotype correlates with fewer straight vessels in their tails. In addition, using a 3D spheroid-based angiogenesis assay^35^ combined with downregulation/upregulation of components of the LMO2 complex, we provide evidence that it plays a pivotal role in endothelial cell migration, tube formation, and continuous tip cell competition during sprouting angiogenesis by transcriptionally regulating downstream target genes such as angiopoietin 2 and VEGF receptors.

## Methods

All methods were carried out in accordance with relevant guidelines and regulations. Biomedical experiments were performed in accordance with the guidelines for Recombinant DNA Experiments (Ministry of Education, Culture, Sports, Science and Technology, Japan). The biomedical experiment committee of Aino University approved all experimental protocols for this study.

### Generation of transgenic mice

LYL1 transgenic mice were produced in our laboratory according to the procedure described by^36^. For the present study, this strain of mouse, B6.Cg-Tg(BOS-mLYL1)/Rbrc (RBRC01839), was provided by RIKEN BRC through the National Bio-Resource Project of the MEXT, Japan. Homozygous mice were obtained through heterozygote intercrossing. Genotyping of each mouse was done using PCR with transgene-specific primers set using tail DNA. The animal experiment committee of the Graduate School of Medicine, Kyoto University and Aino University approved these experiments. This study is reported in accordance with ARRIVE guideline.

### CD31 immunohistochemistry and anatomical analysis

A cryostat (Leica) was used to produce 10 μm-thick sections of fresh-frozen tissue. After fixation in − 30 °C methanol for 10 min, hematoxylin–eosin was used to stain the sections. Immunohistochemistry was then performed on the sections by treatment with 10 μg/ml of anti-CD31 goat polyclonal antibody (R&D systems, AF3628) for 16 h at 4 °C. Whole amount CD31 staining of E8.5 embryos were performed using the same antibody for 24 h at 4 °C. After washing, staining was done using Goat IgG VisUCyte HRP Polymer Detection Reagent (R&D systems) according to the manufacturer’s protocol. Most anatomical analyses and evaluation were done using embryos (E8.5) or 6 weeks old littermate pairs (wild type vs. TG/+) (n = 5). Retinas of 11-week-old littermates pairs (n = 2) were stained with 2 μg/ml of Alexa-Flour 488 conjugated Isolectin GS-IB4 (Thermo Fisher) for 1 h at 4 °C for bifurcation angle analysis.

### Cell culture

Human umbilical vein endothelial cells (HUVECs) and human dermal microvascular endothelial cells (HDMECs) were purchased from PromoCell (PromoCell, Heidelberg, Germany) and cultured in endothelial cell growth medium 2 (EGM2 medium) or endothelial cell growth medium MV2 (MV2 medium) respectively with supplements (PromoCell, Heidelberg, Germany). Cells were used for nucleic acid transfection immediately following a single 1:3 passage. HEK293T (293 T) cells were purchased from Horizon (Horizon, CO) and cultured in glutamax Dulbecco’s modified Eagle’s medium (GIBCO) supplemented 10% bovine fetal serum.

### siRNA transfection

Small interfering RNAs (siRNA) were transfected in HUVECs at 90% confluence by using Lipofectamine RNAiMAX Reagent (Invitrogen, CA, USA) according to the manufacturer’s protocol. The sequences of duplex RNAs were described by^12,29^. eGFP siRNA was used as the transfection control.

### DNA transfection

The whole cDNA sequences of TAL1, LYL1, and β-galactosidase were cloned into the Xba I site in the mammalian expression vector pEF-BOS^37^ to prepare BOSTAL1, BOSLYL1, and BOSβgal, respectively. The empty expression vector pEF-BOS (BOSXba) was used as a transfection control. Each of the DNA sequences was transiently transfected to the cultured HUVECs and HDMECs at 90% confluence using the Lipofectamine 3000 Reagent or jetPEI-HUVEC transfection reagent (Polyplus-transfection S. A., France) for higher transfection efficiency according to the manufacturer’s protocol.

### Real-time qPCR

For the relative expression study, cell lysates were prepared 48 h after siRNA transfection and 72 h after DNA transfection. Following this, cDNA synthesis and quantitative real-time PCR were performed using the CellAmp Direct SYBR RT-qPCR Kit (TaKaRa, Shiga, Japan). Reactions were run on an Eco Real-Time PCR System (Illumina, CA, USA). The signal of each RNA was normalized to that of GAPDH or HPRT1. Oligo. Oligonucleotide primers employed for amplification are listed in the supplemental Table S1. Statistical analysis of the relative expression of each target gene was done after three separate transfections and duplicate PCR reactions (n = 3 × 2).

### Quantitative western blot

HUVEC pellets were lysed in cold lysis buffer (20 mM Hepes/KOH pH7.9, 1 mM EDTA, 400 mM NaCl, 0.5 mM DTT) supplemented with protease inhibitors cocktail (Roche) for each plate 48 h after si-RNA and 72 h after DNA transfection. 10 μg of protein per lane was separated by SDS-PAGE. LMO2, TAL1, LYL1 and GATA2 were detected using anti-LMO2 (Abcam ab222847), anti-TAL1 (Abcam ab155195), anti-LYL1 (Abcam ab229338), and anti-GATA2 (Abcam ab153820) primary antibody. Band intensities of each proteins were quantified with Image-Lab software (Bio-Rad) and compared with those of control HUVEC pellets with si-eGFP or BOSXba transfection. Membranes were reprobed with anti-β-Actin antibody (Abcam, clone AC-15).

### ELISA

Angiopoietin 2 protein levels in cell culture media were quantified by enzyme-linked immunosorbent assay (ELISA), using the Quantikine ELISA human angiopoietin 2 immunoassay (R&D Systems, MN, USA) according to the manufacturer’s protocol. HUVECs were transfected with RNAs or DNAs. Then, 24 h after transfection, the medium was changed and angiopoietin 2 secretion into the culture medium was measured for 24 h. In cases where DNA transfection was performed, the medium was changed 48 h after transfection. Statistical analysis was done after 2 separate transfections and duplicate reactions.

### 3D spheroid-based angiogenesis assay

A 3D spheroid-based angiogenesis assay was performed according to the procedure described by Heiss^35^ with a slight modification. After one passage, HUVECs were plated for siRNA or DNA transfection. Then, 24 h after transfection, cells were suspended in sterile 0.24% w/v methylcellulose solution in EGM2 medium with 10% FBS and aggregated overnight in hanging drops (25 μL) to form cellular spheroids (500 cells/spheroid). Spheroids were embedded into 1 mg/mL bovine acid-treated collagen gels (Nippi, Tokyo, Japan). Vessel sprouting was stimulated with 20 ng/mL (final concentration) VEGF165 (Sigma-Aldrich, MO, USA) 30 min after embedding the spheroids. After a 24 h incubation at 37 °C, spheroids in collagen gels were fixed with 10% paraformaldehyde and in vitro angiogenesis was quantified using cellSens Standard2 software (Olympus, Japan) to measure the cumulative sprout length (CSL) of capillaries, defined by the presence of lumens, that grew from the spheroids after 3 separate HUVEC transfections. When HUVECs or HDMECs were transfected with BOSβgal, whole-mount X-gal staining of spheroids was performed according to the protocol described in^5^.

### Endothelial cell invasion assay

After one passage, HUVECs were plated for DNA transfection. Then, 48 h after transfection, cells were suspended in EGM2 medium with 10% FCS, 1 ng/ml VEGF, and 20 ng/ml bFGF. 5 × 10^4^ cells were plated on BioCoat Matrigel Invasion Chambers (Corning, MA, USA) and the plates were incubated for 24 h in a humidified CO_2_ incubator. After removing non-migrated cells on the upper side of the membrane, migrated cells were fixed with cold methanol (4 °C) for 20 min and stained with 0.1% crystal violet (Sigma-Aldrich, MO, USA).

### Mouse aorta ring assay

Thoracic aortas from 6-week-old mice were dissected from 3 littermate pairs (wild type vs. TG/+) at separate times. After removing all surrounding connective tissue in the medium, they were cut into segments of 1 mm length and placed in Matrigel (Corning, MA, USA). They were incubated in EC growth medium-2 (Promocell, Heidelberg, Germany) containing 10% FBS for 9 days. Aortic explants were then imaged daily by using an inverted microscope (Olympus, Japan). Bifurcation of the sprouting vessels in the explants was quantified by using cellSens Standard2 imaging software (Olympus, Japan) using two pairs of aortic explant culture.

### Dual luciferase assay

pGL3-VEGFR2-enh-pro (VEGFR2-enh-pro) was used as a reporter, in which a firefly luciferase reporter gene was driven by a VEGRF2 promoter and endothelium specific intronic enhancer^38^. In this vector, human VEGFR2 promoter sequence about 500 bp including 5ʹ UTR amplified with primers 5ʹ-CAAAGTTGTTGCTCTGGGATG and 5ʹ-GGGAGCCGGTTCTTTCTCCCA was cloned into multiple cloning site of pGL3Basic vector (Promega) and intronic enhancer sequence about 500 bp was cloned into SalI site of the same vector. A pGL3Basic vector lacking an enhancer or promoter element (Promega) was used as a negative control. pRL-TK (Promega) expressing sea pansy luciferase was used as a control for transfection efficiency. Expression vector (pEF-BOS-LYL1 or pEF-BOS-TAL1) was co-transfected with luciferase reporter vectors into 293 T cells using Lipofectamine 3000 reagent (Invitrogen Life technologies). The luciferase activity was measured 48 h after transfection using the Dual-Luciferase-Reporter Assay System (Promega). Firefly luciferase activity were normalized by sea pansy luciferase ones. Each transfection was carried out in triplicate.

### Statistical analysis

All statistical analyses were performed using Student *t* test.

## Results

### Mice overexpressing LYL1 possess deformed tails

**Figure 1:**
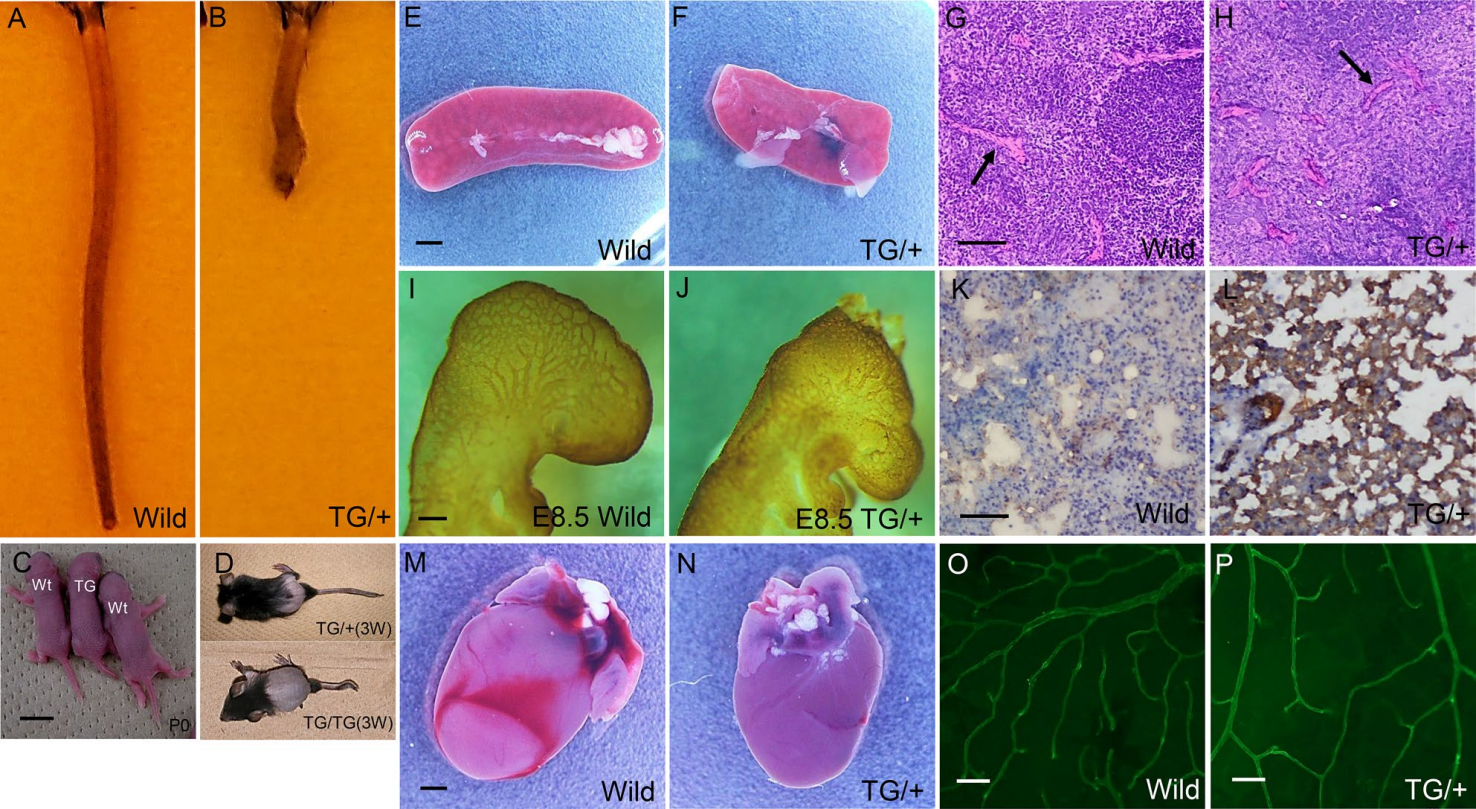
LYL1 transgenic mice have a short tail and round organs. Anatomical features of LYL1 transgenic mice were compared with its wild type littermates macroscopically and microscopically (n = 5). (**A**,**B**) Tail shortening was seen in LYL1 transgenic (TG/ +) mice (**B**) compared with wild type littermates (**A**) at 6 weeks of age. (**C**) The short tail phenotype was observed in the TG/+ P0 mice. Scale bar 5 mm. (**D**) LYL1 transgenic mice showed alopecia later in life, which was severe in homozygotes (TG/TG). (**E**,**F**) The shape of organs in LYL1 transgenic mice was generally rounder and smaller than that of wild type mice. Spleens of 6-week-old TG/+ transgenic mice (**F**) were smaller (about 10% reduction in weight) and rounder than those of wild type littermates (**E**). Bar 2 mm. (**G**,**H**) The peripheral part of the TG/+ mouse spleen showed increased numbers of blood vessels (average 54.2 in TG/+ and 23.7 in wild type littermate in arbitrary field of 4.3 × 4.3 mm in sub-capsular area sections) (*P < 0.05) (indicated by the arrow in GH). They were stained with hematoxylin and eosin. Bar = 20 μm. (**I**,**J**) Representative images of CD31 whole amount immunohistochemistry from E8.5 TG/+ mouse embryo (**J**) and its wild type littermate (**I**). Note the hyperbranching blood vessels and relative lack of straight vessels in peripheral part of brain of TG/+. Bar 50 μm. (**K**,**L**) CD31 (PECAM) staining of a lung section from a TG/+ mouse (**L**) and its wild type littermate (**K**). Smaller alveolar spaces with smaller diameters (mean value 39.2 μm in wild type and 23.4 μm in TG/+) were observed in TG/+ lungs (**L**) (**P < 0.01 n = 30). In TG/+ lungs, smaller vessels and capillaries surrounding alveoli were more positively stained (brown color) than those of the wild type littermate. Bar 100 μm. (**M**,**N**) In the cardiovascular system, a smaller and rounder heart was seen in TG/+ mice (**N** about 10% reduction of weight and axis length) compared with hearts in wild type littermates (**M**). Bar 2 mm. (**O**,**P**) TG/+ blood vessels showed wider branching angles (average 79.6°in TG/+ , 61.8°in wild type littermate) (**P < 0.01) typical of those at the 11-week-old mouse retina stained with Alexa-Flour 488 Isolectin IB4 (**P**) compared with those from wild type littermates (**O**). Bar = 50 μm.

A tail shortening phenotype was observed in transgenic mice that overexpressed the basic helix-loop-helix transcription factor LYL1 driven by the promoter of the human elongation factor gene (Fig. 1A,B). The foundation and maintenance of these transgenic mice was described in our previous report^36^. These mice possessed short and deformed tails at birth (Fig. 1C), organs with a round shape that were about 10% lower in weight and length than their wild type littermates at 6 weeks of age. Later in life these mice exhibited marked alopecia (Fig. 1D) and developed malignant lymphoma as described in Ref.^36^. Spleens of 6-week-old TG/+ mice have a round shape and are smaller than those of their wild type littermates that usually exhibit dumbbell-shaped spleens (Fig. 1E,F). They also exhibited increased blood vessel density (Fig. 1G,H). The whole amount CD31 immunohistochemistry of E8.5 TG/+ mouse embryos (Fig. 1J) were also characterized hyperbranching of brain vessels at the peripheral part of brain and relative paucity of straight brain vessels when compared with their wild type littermates (Fig. 1I). The result of bifurcation point counting at the peripheral part of brains was shown in Supplemental Fig. S1. Statistically significant (**P < 0.01) increase in branching points was seen in TG/+ mouse embryos. Especially in the lung, which is organized by two closely related branched anatomical systems (tracheal tubes and blood vessels), TG/+ mice were observed to have smaller alveolar spaces and capillaries than their wild type littermates at 6 weeks of age. Immunohistochemical analysis showed alveolar capillaries to be more strongly positive for CD31 in TG/+ mice at 6 weeks of age than those of wild type littermate (Fig. 1 K,L). Mean alveolar diameter in the sub-pleural area of TG/+ mice was 23.4 μm ± 12.6 SD and that of wild type littermates 39.2 μm ± 17.1 SD (**P < 0.01, n = 30). In the cardiovascular system, the heart and large vessels of 6-week-old TG/+ mice was smaller (about 10% reduction in weigh, 0.112 g in TG/+ (BW = 16.5 g) and 0.138 g in the wild type littermate (BW = 18.5 g) representatively, length, and diameter) than those of wild type litter mates (Fig. 1M,N). Differences in vessel branching were observed in 11-week-old TG/+ mice at the surface of the retina, in which they generally had a wider branching angle than those of their wild type littermates (Fig. 1O,P). Mean branching angle of TG/+ mice was 79.6° and that of wild type retina 61.8° (**P < 0.01) (Supplemental Fig. S2). All these anatomical findings suggest that the reduced formation of straight blood vessels during tail development might be responsible for the short and deformed tail phenotype of the transgenic mice, which could be closely associated with the hyperbranching of the vascular system and resulting hypervascularity in the peripheral part of all organs.

### Capillaries sprouting from aortic walls of LYL1 transgenic mice exhibited an increased number of bifurcations

**Figure 2:**
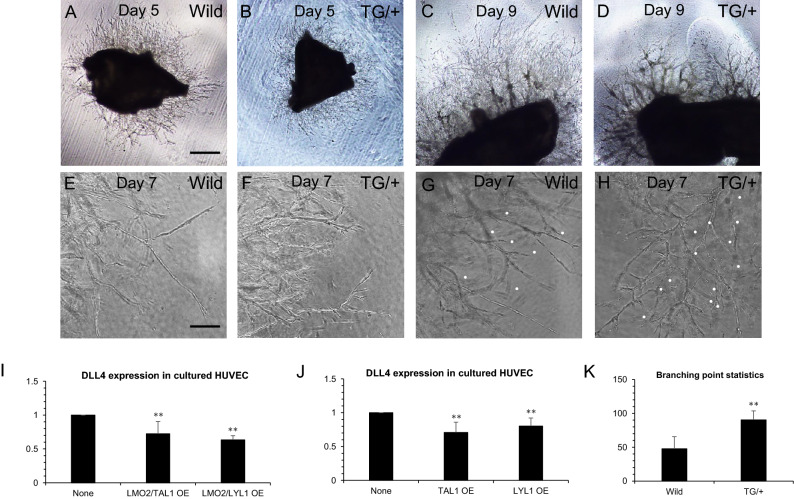
LYL1 transgenic mouse aorta explants cultured in Matrigel showed hyperbranching of sprouting capillaries in the aorta ring assay. Three paired aorta ring sections were dissected out at separate times from 6-week-old wild type littermates and LYL1 transgenic mice, embedded in Matrigel, and cultured until day 9. (**A**,**B**) Representative low magnification images of aorta ring culture of wild type littermates (**A**) and LYL1 transgenic (TG/ +) mice (**B**) at day 5. Capillary sprouting was slightly delayed in TG/+ aortas compared with wild type controls. Note the increased density of capillaries near the TG/+ aortic wall in (**B**). (**C**,**D**) Representative low magnification images of other paired cultures at day 9, by which remodeling of capillaries has begun. In TG/+ aorta culture (**D**), remodeled vessels showed higher frequencies of branching, which resulted in a relative decrease in the number of straight capillaries at the periphery compared with wild type aorta ring culture (**C**). (**E**,**F**) Representative high magnification images of other paired (**E**: wild type, **F**: TG/ +) aorta ring assays at day 7, in which capillaries from TG/+ aortas showed hyperbranching. (**G**,**H**) Representative high magnification images of another paired (**G** wild type, **H** TG/ +) aorta ring assay at day 7. Unambiguous bifurcations were indicated by white dots. (**I**) Relative expression level of DLL4 after double transfection of the expression vectors BOSLMO2/BOSTAL1 or BOSLMO2/BOSLYL1 into cultured HUVECs. Statistically significant (**P < 0.01) decreases in DLL4 expression compared with control HUVECs were seen after both combined transfections. (**J**) Relative expression levels of DLL4 after a single transfection of the expression vector BOSTAL1 or BOSLYL1 into cultured HUVECs. Statistically significant (**P < 0.01) decreases of DLL4 compared with control HUVECs were seen after both single transfections. K. A comparison of average clear bifurcation points per 11 arbitrary fields (each 10 different z-axis points) at the same distance from the aorta showed that there were 48 in wild type and 90 in TG/+ aortas (**P < 0.01). Bar 1250 μm (**A**–**D**), Bar = 200 μm (**E**–**H**).

In order to observe vascular branching patterns of the LYL1 transgenic mice in vitro, aorta ring sections about 1 mm wide were freshly prepared from 6-week-old LYL1 transgenic mice and their wild type littermates and implanted into Matrigel or type 1 collagen gel and cultured until day 9 with endothelial cell growth medium 2 (EGM2) supplemented with several growth factors and 10% FBS in a CO_2_ incubator. In this aorta ring assay, slightly delayed budding of capillaries from the aortic wall was seen in 6-week-old TG/+ mice. From the beginning of budding, formation of straight capillary tubes was greatly reduced in TG/+ mice. In order to estimate the LYL1 expression level in endothelia of aortic explants of TG/+ mouse, mRNA was isolated from aortic cultures of TG/+ and its wild type littermate at days 5 and 7. At day 5, 101.14 times mRNA level was detected in TG/+ explant compared with the wild type littermate. At day 7, 120 times mRNA level in TG/+ explant in qPCR analysis. Capillaries from LYL1 transgenic mouse aortas showed an increased number of bifurcations that resulted in vessel hyperdensity near the aortic wall (Fig. 2A,B). This hyperbranching phenotype of sprouting vessels was also seen in day 9 culture when maturation and reorganization of sprouting capillaries had already begun (Fig. 2C,D). The counting of unambiguous bifurcation points was done at day 7. In wild type mouse aorta ring cultures, the average number of branching points (bifurcations) per 11 arbitrary fields (1 field: 1.05 × 1.4 mm) at the same distance from aorta ring was 48, while that of LYL1 transgenic mice was 90 (n = 11 each for wild type and TG/+) (**P < 0.01) (Fig. 2E–H,K). The branching pattern of blood vessels is largely determined by VEGFR-DLL4-Notch^39^, and the oligomeric transcription factor consisting of TAL1, LMO2, LDB2, GATA2 was recently shown to be involved in regulation of VEGF-induced DLL4 upregulation in sprouting tip cells^30^. We speculated that forced overexpression of the LYL1 transcription factor (which also binds to LMO2) in endothelial cells affects DLL4 expression levels in endothelia by forming an aberrant transcription factor complex in the Dll4 promoter/enhancer. Since expression of DLL4 from the aortic wall prior to culture was very low, and that from aortic culture after day 5 was greatly influenced by the number of tip cells (DLL4 is the tip cell marker protein), we transfected the cultured HUVECs with the expression vector of LYL1 (EF-BOS-LYL1) singly or combined with LMO2 and evaluated the expression level of DLL4 at 48 h after transfection by qPCR. A reduction of about 40% in DLL4 expression was observed for LMO2/LYL1 double transfection and 20% for LYL1 single transfection (Fig. 2I,J). In addition to LYL1 overexpression, TAL1 overexpression also showed similar downregulation of DLL4. In mice of DLL4 haploinsufficiency, the hyperbranching phenotype was seen in hindbrain vessels and in a postnatal angiogenesis assay of P4-6 retina^40^.

### Downregulation of all members of the LMO2 and TAL1 transcription factor complex except LYL1 strongly inhibits sprouting angiogenesis in vitro

**Figure 3:**
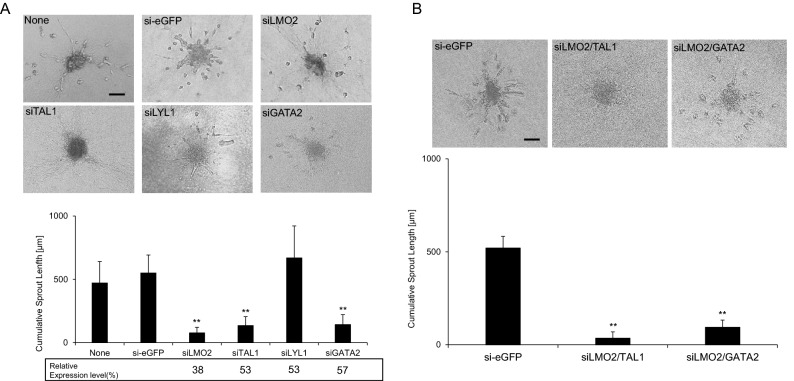
Knockdown of LMO2, TAL1, and GATA2 leads to inhibition of endothelial sprouting and tube formation in vitro*,* but LYL1 knockdown does not. Knockdown of components of the LMO2 complex was achieved using siRNA with HUVEC spheroids. (**A**) Representative images from 3D spheroid-based in vitro angiogenesis assays and a statistical summary of CSL measurements from spheroids. Cultured HUVECs were transfected with siRNAs of eGFP, LMO2, TAL1, LYL1, and GATA2 as indicated in the figure. siRNA of eGFP was used as a transfection control. Relative mRNA levels (average of 2 separate transfections) of HUVECs transfected with each siRNA compared with that of si-eGFP-transfected cells are indicated below the names of the RNAi target genes below the statistical summary of CSL measurements. Note that sprouts with continuous tubes from the spheroids are counted in this analysis. Statistically significant (**P < 0.01) decreases in CSL were observed in spheroids transfected with siRNAs for LMO2, TAL1, and GATA2. (**B**) Cultured HUVECs were transfected with siRNAs of eGFP, LMO2 combined with TAL1, and LMO2 combined with GATA2 as indicated in the figure. siRNA of eGFP was used as a transfection control. Statistically significant (**P < 0.01) decreases in CSL were observed in spheroids transfected with LMO2/TAL1 and LMO2/GATA2 siRNAs (combined transfection). Combined knockdowns of LMO2/TAL1 and LMO2/GATA2 almost abolished sprouting from spheroids. Bar 200 μm.

In order to explore the role of the transcription factor consisting of LMO2 and TAL1 in vessel sprouting and subsequent tube formation in vitro, we studied the efficiency of capillary sprouting induced by VEGF-A in spheroids of cultured HUVECs in type 1 collagen gel culture. This 3D spheroid-based angiogenesis assay was used to assess the effect on tube formation of downregulation by siRNAs of LMO2 transcription factor complex members (LMO2, TAL1, LYL1, and GATA2). Prior to the assay, expression of each transcription factor was estimated by qPCR (Fig. 3A: relative expression level) and quantitative Western blot. At mRNA levels, expression level is reduced to 38%, 53%, 53%, and 57% compared with si-eGFP transfection and at protein levels, 25%, 33%, 35%, and 40% by transfection of si-LMO2, si-TAL1, si-LYL1, and si-GATA2 respectively. Each spheroid of 500 HUVECs containing transfected siRNA was formed in methylcellulose and then transferred to a type 1 collagen gel to assess its capacity for vessel sprouting. After stimulation of VEGF-A at a final concentration of 20 ng/ml in culture medium for 24 h, the total length of capillary sprouts with lumens was counted under a microscope. Only sprouts that were continuous from the spheroid to the tip cells were counted and compared with those that were transfected with control siRNA (si-eGFP). As shown in Fig. 3A, the cumulative length of the vessel sprouts decreased to 14%, 25%, and 26% of control (eGFP) by transfection with LMO2, TAL1, and GATA2 siRNAs, respectively. In contrast, and most importantly, it was not influenced by transfection of LYL1 siRNA.

### Combined downregulation of LMO2/TAL1 and LMO2/GATA2 further inhibits sprouting angiogenesis

The synergic effect of downregulation via knockdown (KD) of members of LMO2 and TAL1 transcription factor complex was assessed using the 3D angiogenesis assay described above. As shown in Fig. 3B, sprouting angiogenesis was almost completely abolished in both the LMO2 combined with TAL1 and LMO2 combined with GATA2 KDs. The latter result was in accordance with a previous report^28^.

### Angiopoietin 2 secretion levels correlate with sprout length and endothelial migration

**Figure 4:**
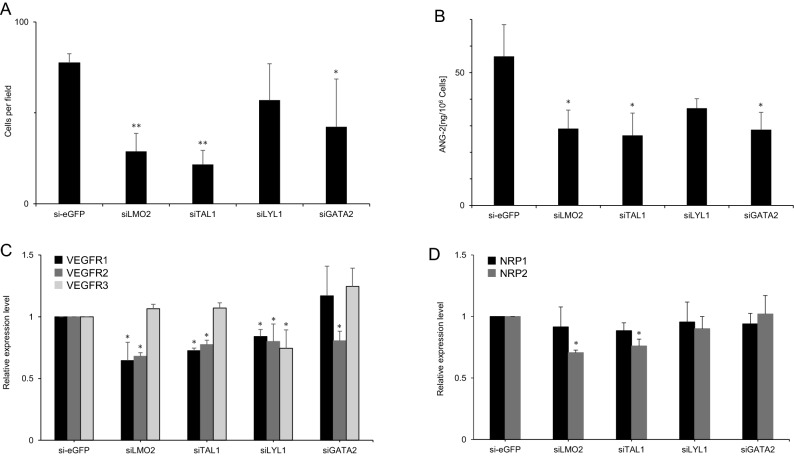
Effects of LMO2, TAL1 silencing on endothelial cell migration and their downstream targets’ expression. Knockdown of components of the LMO2 complex was achieved using siRNA with cultured HUVEC. (**A**) Number of invading HUVECs transfected with siRNA per field is indicated in the figure. Cell counting was done in four randomly selected fields after 2 separate DNA transfections. Invasion of LMO2, TAL1 knockdown HUVECs into a Matrigel extracellular matrix was significantly (**P < 0.01) reduced compared with the transfection control (si-eGFP). (**B**) Cultured HUVECs were transfected with the indicated siRNA. Angiopoietin 2 (ANG-2) secretion into the culture medium after 24 h incubation was measured by ELISA. Statistically significant (*P < 0.05) decreases in ANG-2 secretion were observed in culture media obtained from si-LMO2-, si-TAL1-, and si-GATA2-transfected HUVECs, but not in that from si-LYL1-transfected HUVECs. (**C**,**D**) Cultured HUVECs were transfected with siRNA of LMO2, TAL1, LYL1, GATA2, and the transfection control si-eGFP. Relative mRNA expression levels of VEGFR1, VEGFR2, VEGFR3 (**C**), and NRP1, NRP2 (**D**) compared with those of si-eGFP transfection controls are shown. They were measured by real-time qPCR after normalization using GAPDH as an internal reference. Statistically significant (*P < 0.05) decreases are indicated.

Angiopoietin 2 has been identified as a downstream transcriptional target molecule of TAL1, LYL1, and LMO2^29^. Angiopoietin 2 is expressed at high levels in tip cells and plays two important roles in sprouting angiogenesis. Firstly, it liberates endothelial cells by detachment of mural cells^39^. Secondly, it binds to integrins on endothelial cells such as tip cells that express low levels of Tie2 and stimulates migration^34^. Migration of HUVECs into matrigel was measured after knockdown of LMO2, TAL1, LYL1, and GATA2. When LMO2 and TAL1 were downregulated in HUVECs, the number of cells that migrated into Matrigel significantly decreased (**P < 0.01) when compared with control si-eGFP-transfected cells (Fig. 4A). In parallel with the 3D angiogenesis assay, angiopoietin 2 secreted by HUVECs into the culture medium was measured by ELISA after 24 h. Generally, angiopoietin 2 protein levels in the culture medium were reduced by downregulation of each member of the LMO2 transcription factor complex. As shown in Fig. 4B, the quantity of protein decreased to 52%, 47%, 65%, and 51% of that in the control (eGFP) by transfection of LMO2, TAL1, LYL1, and GATA2 siRNA, respectively. Angiopoietin 2 levels in the supernatant showed a strong correlation with CSL in spheroids treated with each siRNA (correlation coefficient: r = 0.71) and number of cells per field in endothelial cell invasion assay of HUVEC treated with each siRNA (r = 0.92) (Supplemental Fig. S3). We concluded that the reduced sprout length and endothelial cell migration resulting from knockdown of LMO2 and TAL1 can at least partially be explained by lowered secretion of angiopoietin 2 from tip cells.

### Downregulation of LMO2 or TAL1 suppresses expressions of the main VEGF receptors and co-receptors

In search of other downstream targets of the LMO2 transcription factor complex that are responsible for the inhibition of sprouting angiogenesis, we quantified the mRNA levels of the main VEGF receptors (VEGFR1, VEGFR2, and VEGFR3) and their co-receptors (neuropilin 1 and neuropilin 2) after downregulation of LMO2 transcription factor complex members. The mRNA levels of VEGFR1, VEGFR2, and neuropilin 2 (NRP2) significantly decreased after LMO2 or TAL1 knockdown (Fig. 4C,D). In the case of GATA2 knockdown, only the VEGFR2 mRNA level decreased.

### LYL1 overexpression inhibits endothelial cell migration and tube formation in sprouting angiogenesis

**Figure 5:**
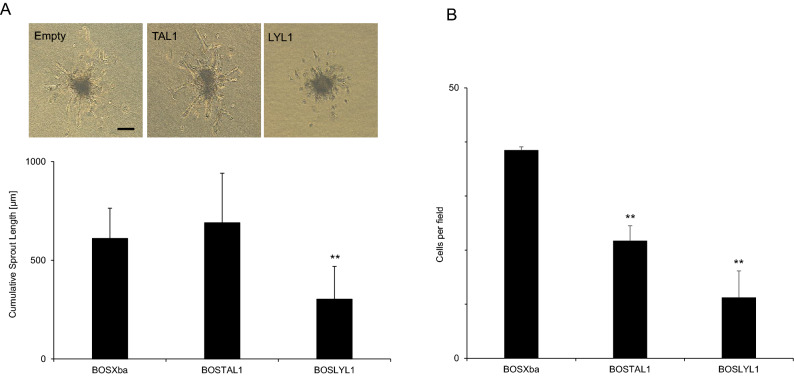
Overexpression of LYL1 inhibits endothelial cell migration and sprouting angiogenesis in a 3D spheroid-based assay. Overexpression of TAL1 or LYL1 was achieved using DNA transfection with HUVEC spheroids. (**A**) Cultured HUVECs were transfected with mammalian expression vectors of TAL1 and LYL1. The empty mammalian expression vector BOSXba was used as a transfection control. Statistically significant (**P < 0.01) decreases in CSL were observed in spheroids transfected with BOSLYL1. Representative images of the analyses are also shown. (**B**) Number of invading HUVECs (per field) transfected with expression vectors are indicated in the figure. Cell counting was done in four randomly selected fields after 2 separate DNA transfections. Invasion of TAL1, LYL1 OE HUVECs into Matrigel extracellular matrix was significantly (**P < 0.01) reduced compared with the transfection control (BOSXba). Bar 200 μm.

In order to estimate the effect of TAL1 and LYL1 overexpression (OE) on endothelial cell migration and tube formation in sprouting angiogenesis, we transfected the mammalian expression vectors, BOSTAL1 or BOSLYL1, into cultured HUVECs. After confirming overexpression of TAL1 or LYL1 in cultured HUVECs (5 and 3 times upregulation using real-time qPCR and 10 and 8 times upregulation using quantitative Western blot compared with control BOSXba empty vector transfection by BOSTAL1 and BOSLYL1 transfection respectively), transfected cells were used for 3D angiogenesis assays (Fig. 5A). The same assay as described above was used and sprouting from spheroids was stimulated by VEGF-A at a final concentration of 20 ng/ml. After incubation for 24 h, CSL in transfected HUVECs was measured under a microscope and compared with those of control cells that had been transfected with BOSXba empty vectors. As shown in Fig. 5A, CSL from the spheroids of the BOSLYL1-transfected HUVECs decreased to 50% of that of the control EF-BOS empty vector (BOSXba)-transfected HUVECs. Thus, increased expression of LYL1 in HUVECs inhibits sprouting angiogenesis. In contrast, increased expression of TAL1 in HUVECs did not affect vessel sprouting (Fig. 5A). Since vessel sprouting is affected by endothelial migration capacity, an endothelial invasion assay was performed. It was reported that TAL1 overexpression in endothelium inhibited endothelial migration^7^. In our assay, the number of cells that migrated into Matrigel significantly decreased when TAL1 or LYL1 was overexpressed in HUVECs as compared with control (Fig. 5B). Therefore LYL1 overexpression resulted in a greater reduction in endothelial migration than did TAL1 overexpression.

### TAL1-expressing cells preferentially occupy the tip cell position in vessel sprouts

**Figure 6:**
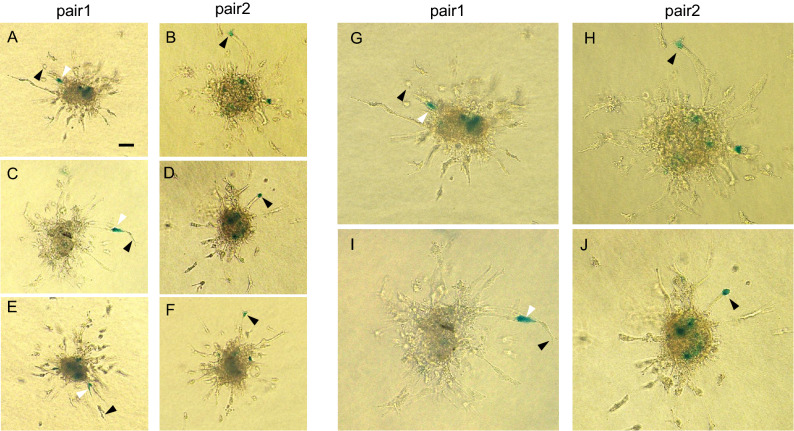
TAL1-expressing cells preferentially occupy the tip position over LYL1-expressing cells in vessel sprouts in vitro. Cultured HUVECs were transfected with a mammalian expression vector encoding LYL1, TAL1, and β-galactosidase. In pair1, non-labeled TAL1-expressing cells were mixed at 1:1 ratio with β-galactosidase-labeled LYL1-expressing cells. In pair2, β-galactosidase-labeled TAL1-expressing cells were mixed at 1:1 ratio with non-labeled LYL1-expressing cells. Both chimeric spheroids were cultured in type 1 collagen gel for 24 h with 20 ng/ml VEGF-A stimulation. Labeled cells were detected by whole mount X-gal staining immediately after culture. Cells at the tip position in the sprouts are indicated by black arrowheads and those at the stalk position by white arrowheads. (**A**,**C**,**E**,**G**,**I**) Representative images of X-gal-stained pair1 spheroids. The labeled cells (LYL1-expressing cells in this pair) were preferentially located in the stalk position. (**B**,**D**,**F**,**H**,**J**) Representative images of X-gal-stained pair2 spheroids. The labeled cells (TAL1-expressing cells) tend to occupy the tip position. The distributions of labeled cells in all chimeric spheroids are shown in Table 1. Bar 100 μm.

In order to explore the effect of the components of LMO2 transcription factors in endothelial cells on the competition between tip and stalk cells in vessel sprouts, we performed 3D spheroid-based angiogenesis assays using chimeric spheroids of TAL1-expressing and LYL1-expressing HUVECs. To achieve higher labeling efficiency, we used a linear polyethyleneimine based jet PEI-HUVEC transfection method (Polyplus-transfection S.A., France) throughout this experiment. About 70% of HUVECs were strongly positive and 15% were weakly positive in X-gal staining after the transfection of BOSβgal. Labeling of HUVECs was done by co-transfection of BOSβgal and the expression vector of each transcription factor. Two types of chimeric spheroid were prepared by 1:1 ratio mixing of cells after DNA transfection before spheroid formation in hanging drops. One type (pair1) consisted of non-labeled TAL1-expressing cells, obtained by single transfection of BOSTAL1, and β-galactosidase-labeled LYL1-expressing cells. The other type (pair2) consisted of β-galactosidase-labeled TAL1-expressing cells and non-labeled LYL1-expressing cells. Both pair1 and pair2 chimeric spheroids were stimulated by 20 ng/ml VEGF-A in type 1 collagen gel culture for 24 h. The number and position of labeled endothelial cells in vessel sprouts was detected by whole mount X-gal staining. As shown in Fig. 6 and Table 1, among a total of 77 pair1 chimeric HUVEC spheroids, in only 1 case did LYL1-expressing cells occupy the tip position in sprouts growing from the spheroid. In contrast, the tip position was occupied by TAL1-expressing cells in 6 out of a total of 78 pair2 chimeric spheroids. The stalk position was occupied by LYL1-expressing cells in 10 out of 77 pair1 chimeric spheroids, while it was occupied by TAL1-expressing cells in only 2 out of 78 pair2 chimeric spheroids (**P = 0.0018). This result shows that TAL1-expressing cells preferentially occupy the tip position in vessel sprouts while LYL1-expressing cells are preferentially located in the stalk position.

**Table 1:** Number of HUVEC spheroids in which the labeled cells occupied the indicated positions after sprouting in collagen gel.

	Pair1 spheroids	Pair2 spheroids
Tip position	1	6
Stalk position	10	2
Internal position	66	70
Total	77	78

**P = 0.0018.

Similar experiment was also performed using human dermal microvascular cells (HDMECs). In this experiment, to facilitate the spheroid formation and to inhibit apoptotic microvascular endothelial cell death during the procedure of sprouting in type 1 collagen gel, we mixed HDMECs (labeled) and HUVECs (non-labeled) at 4:1 before overnight hanging drop to form spheroids. This mixing increased the sprouting ratio from 25 to 50%. Because only HDMECs were labeled, we can identify all X-gal positive cells are derived from HDMECs. As shown in Supplemental Fig. S4 and Table 2, among a total of 32 pair1 chimeric HDVEC spheroids, no LYL1-expressing cells occupied the tip position in sprouts growing from the spheroid. In contrast, the tip position was occupied by TAL1-expressing cells in 2 out of a total of 18 pair2 chimeric spheroids. The stalk position was occupied by LYL1-expressing cells in 5 out of 32 pair1 chimeric spheroids, while it was not occupied by TAL1-expressing cells (none out of 18 pair2 chimeric spheroids) (**P = 0.0078). This result shows that TAL1-expressing cells preferentially occupy the tip position in vessel sprouts while LYL1-expressing cells are preferentially located in the stalk position in HDMEC spheroids as well.

**Table 2:** Number of HDMEC spheroids in which the labeled cells occupied the indicated positions after sprouting in collagen gel.

	Pair1 spheroids	Pair2 spheroids
Tip position	0	2
Stalk position	5	0
Internal position	27	16
Total	32	18

**P = 0.0078.

### VEGFR1 and VEGFR2 expression are differentially regulated by TAL1 and LYL1

**Figure 7:**
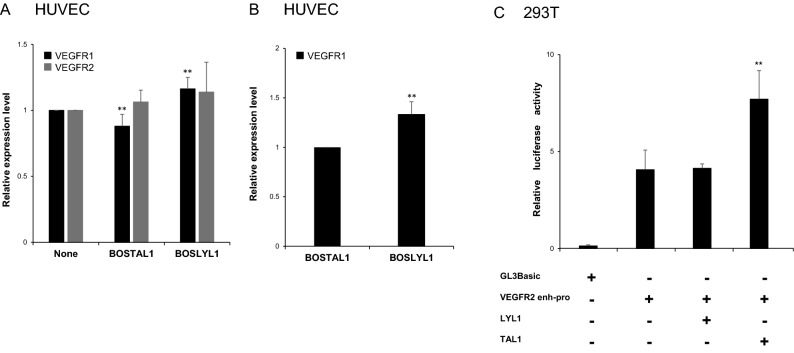
Differential VEGFR1 expression with TAL1 or LYL1 overexpression. Cultured HUVECs were transfected singly with the mammalian expression vectors encoding TAL1 (BOSTAL1) or LYL1 (BOSLYL1). After 48 h of DNA transfection, mRNA was prepared for RT-PCR based quantification of mRNA levels of VEGFR1 and VEGFR2 using HPRT1 as an internal reference. Statistical analysis of the relative expression levels of both mRNAs was carried out after 3 separate transfections and dual real-time PCR reactions (n = 3 × 2). (**A**) A statistically significant (**P < 0.01) decrease in VEGFR1 expression level was observed after BOSTAL1 transfection while an increase was seen after BOSLYL1 transfection. (**B**) An approximately × 1.3 upregulation of VEGFR1 expression levels was observed in response to LYL1 overexpression compared with TAL1 overexpression. C. Effect of LYL1 or TAL1 on VEGFR2 enhancer-promoter. A firefly luciferase reporter vector driven by a VEGFR2 enhancer-promoter (VEGFR2 enh-pro) was transfected into 293 T cells with vectors expressing LYL1 or TAL1 in various combinations. VEGFR2 enhancer and promoter activities are presented as a ratio of firefly luciferase activity to sea pansy luciferase activity. GL3Basic is a promoter-less vector, which was used as a negative control.

In order to explore the molecular mechanism specifying the location of TAL1- and LYL1-expressing cells in vessel sprouts, we quantified the mRNA levels of VEGFR1 and VEGFR2 by RT-PCR after transfection of the expression vectors BOSTAL1 and BOSLYL1 into HUVECs. These transfections were done in the same way as the 3D spheroid-based angiogenesis assay using chimeric spheroids and achieved more than 10 times upregulation of both transcription factors compared with non-transfected HUVECs. mRNA levels were measured and analyzed after 3 separate dual DNA transfection and PCR reactions (n = 3 × 2). In BOSTAL1-transfected HUVECs, the relative expression level of VEGFR1 was suppressed to 88% of that in non-transfected cells (**P < 0.01), while it increased to 116% in BOSLYL1-transfected HUVECs (**P < 0.01) (Fig. 7A). This result means that about a 1.3-times upregulation of VEGFR1 expression (**P < 0.01) was seen in LYL1-expressing cells compared with that in TAL1-expressing cells (Fig. 7B). No statistically significant upregulation or downregulation of VEGFR2 mRNA levels resulted from transfection of BOSTAL1 and BOSLYL1 in cultured HUVEC system. We further explored the fine regulation of VEGFR2 expression by LYL1 or TAL1 transcription factors using luciferase reporter with VEGFR2 intronic enhancer sequence having TAL1 binding sites in it (pGL3 VEGFR2 enh-pro) in dual luciferase assay. This sequence is responsible for endothelium specific expression of VEGFR2 in mouse^38^. Overexpression of LYL1 achieved by co-transfection of LYL1 expression vector in 293 T cells results in the similar luciferase activity as endogenous one. On the other hand, TAL1 OE significantly (about 2 times) enhance the relative luciferase activity (Fig. 7C).

According the results of Jakobsson et al., differential VEGFR levels affect tip cell selection and VEGFR1-downregulated cells (the TAL1-expressing cells in our experiment) dominated in the tip cell population in the assay using mosaic embryoid bodies^41^. Although VEGFR2 expression was not affected by TAL1 upregulation in cultured HUVECs, our luciferase assay data indicated that rapid upregulation of VEGFR2 could be achieved by TAL1 expression though its intronic enhancer probably by chromatin remodeling in the tip cells. We concluded that VEGFR2 upregulation combined with VEGFR1 downregulation by TAL1 OE and VEGFR1 upregulation by LYL1 OE in endothelial cells affects tip cell selection in vessel sprouts and results in TAL1-expressing cells preferentially locating to the tip cell position in the sprouts. Since VEGFR1 can function as a VEGF-A scavenging receptor^42,43^, this result is likely to be associated with the inhibition of endothelial migration seen in Fig. 5.

## Discussion

The basic helix-loop-helix proteins TAL1, LYL1 and the LIM-only protein LMO2 were identified from the breakpoints of chromosomal translocations associated with T cell acute leukemia. Among these three factors, both TAL1 and LMO2 are necessary for the generation of hematopoietic stem cells because the TAL1- and LMO2-knockout mouse phenotypes are bloodless. Formation of the canonical oligomeric transcription factor complex consisting of E2A-TAL1-LMO2-LDB1-LMO2-GATA2 in the nucleus of hemogenic endothelia is considered to be essential for the initiation of adult type hematopoiesis. Overexpression of both TAL1 and LMO2 in T cells results in leukemogenesis by formation of aberrant transcription factors such as E2A-TAL1-LMO2-LDB1-LMO2-TAL1-E2A that disrupt normal differentiation of T cells. Similarly, overexpression of LYL1 also produces leukemia/lymphoma in mice by trapping and inhibiting E2A proteins^36^. Aberrant expression of members of the LMO2 transcription factor complex results in defects in differentiation of blood cells that eventually lead to leukemogenesis^44^.

With respect to cardiovascular development, LMO2, TAL1, and LYL1 are endothelium-specific transcription factors. The overexpression of the LYL1 transcription factor in endothelia of 6-week-old LYL1 transgenic mice results in increased vessel branching in the aorta ring assay and hyper vascularity in the peripheral regions of organs. Its short tail phenotype is associated with a relative paucity of straight vessels during embryonic development. We conclude that these findings are due to reduced expression of DLL4 in tip cells as a result of overexpression of LYL1 disrupting the transcription factor complex consisting of LMO2 and TAL1 that acts on the DLL4 promoter. TAL1 overexpression in cultured HUVEC results in similar downregulation of DLL4 probably by forming the aberrant transcription factor complex such as TAL1-LMO2-TAL1. Qutub and Popel proposed a multiscale systems model that closely simulated the mechanism underlying sprouting angiogenesis and analyzed the effect of DLL4 haploinsufficiency on the pattern of vessel growth^45^. In accordance with our findings, vessel length is greater but more variable in DLL4^+/−^-knockout mice compared with those in simulated controls, which is a result of increased numbers of sprout tips and more branching.

In in vitro spheroid-based angiogenesis assays combined with targeted gene knockdown using RNAi, siRNA that targeted LMO2, TAL1, and GATA2 inhibited sprouting angiogenesis whereas silencing LYL1 did not. The synergistic effect of LMO2/TAL1 and LMO2/GATA2 on vessel sprouting observed here, as well as in a previous study^28^, strongly suggests that LMO2 acts as a member of a multimeric transcription factor complex. This positive role of the LMO2 transcription factor complex in vessel sprouting is partly explained by upregulation of a downstream direct target, angiopoietin 2, because its levels in supernatant from cultured endothelium showed a strong correlation with CSL. The presence of a sequence consisting of the E-box-11 bp-GATA binding site in the angiopoietin 2 promoter proximal region strongly suggests that the LMO2 transcription factor complex consisting of TAL1-LMO2-GATA2 directly binds to this site and positively regulates angiopoietin 2 expression^29^. Among the main VEGF receptors, VEGFR2 (Flk-1) was first identified as a TAL1 and GATA2 downstream target and is considered the VEGF receptor that most influences sprouting angiogenesis. Its enhancer has both the TAL1 and GATA binding sites^20^. Since our data showed that LMO2 also positively regulates its expression, the most likely conformation of the transcriptional regulators is TAL1-LMO2 or LMO2-GATA2, in which LMO2 can play a stabilizing role in the complex. Coma et al. showed that the VEGF co-receptor neuropilin 2 is a direct transcriptional target of the LMO2-GATA2 complex^28^. There is a GATA binding site in its promoter region that positively regulates its expression upon LMO2-GATA2 binding. Our data showed that a single LMO2 knockdown downregulated its mRNA level, suggesting that LMO2 acts to stabilize the protein complex. Thus, the LMO2 transcription factor complex (TAL1-LMO2, LMO2-GATA2, and TAL1-LMO2-GATA2) plays a positive role in sprouting angiogenesis by upregulating angiopoietin 2 and VEGFR2 with the involvement of neuropilin 2 in our VEGF-A (VEGF165)-induced sprouting angiogenesis assay. Our study shows that VEGFR1 and VEGFR2 are also regulated by TAL1, LYL1, and LMO2. A recent study showed that there is a substantial reduction in the expression of 33 core angiogenesis genes, out of the 84 studied, after LMO2 knockdown^46^. Among which, sphingosine kinase 1 (SphK1) was identified as another downstream target of the transcription factor complex consisting of LMO2/TAL1. SphK1 is involved in generation of sphingosine 1 phosphate (S1P), an intracellular second messenger, which plays a key role in endothelial cell migration^47^. Further studies are needed to determine how expression levels of each downstream gene contribute to vessel sprouting.

Forced LYL1 expression in cultured endothelial cells also inhibited subsequent VEGF-A-induced sprouting angiogenesis in the 3D spheroid-based angiogenesis assay. This inhibition is likely to be related to the reduced endothelial migration of LYL1-expressing endothelial cells. VEGFR1, which has a higher affinity for VEGF-A but lower tyrosine kinase activity in its intracellular domain, functions as a scavenger receptor of VEGF-A and negatively regulates VEGF-A-induced endothelial migration in sprouting angiogenesis. In its promoter region, there is a CREB binding site and CREB binding to it positively regulates its expression^48^. LYL1, but not TAL1, can bind to CREB to form a heterodimer^49^. Indeed, LYL1 overexpression resulted in VEGFR1 upregulation while TAL1 overexpression downregulated VEGFR1 expression in our study using cultured HUVECS. LYL1 overexpression and a corresponding VEGFR1 upregulation in endothelial cells could explain the decrease in endothelial migration and in vitro sprouting angiogenesis. According to the results of Jakobsson et al., tip cells are continuously competing for position during sprouting angiogenesis and differential VEGFR1 or VEGFR2 levels affect tip cell selection^41^. VEGFR2 upregulation combined with VEGFR1 downregulation in TAL1-expressing cells and its upregulation in LYL1-expressing cells might give TAL1-expressing endothelial cells an advantage in this competitive positioning in our sprouting angiogenesis assay using 1:1 mixed endothelial cells. This sprouting inhibition by LYL1 OE is consistent with the vascular stabilizing effects of LYL1 described in a previous report^14^.

**Figure 8:**
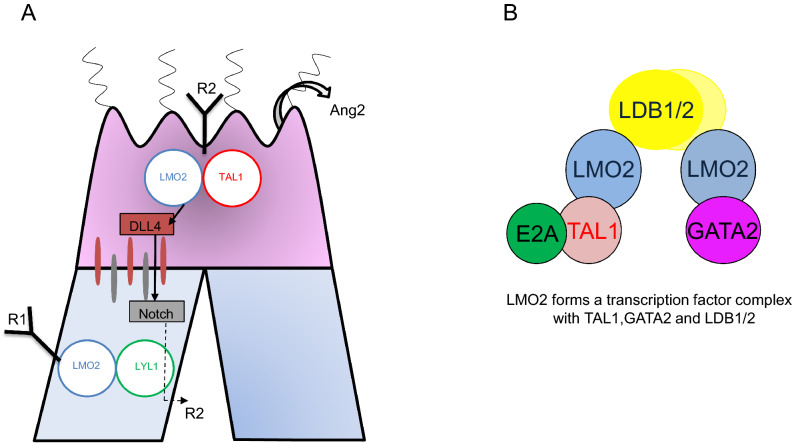
Hypothetical mechanism of transcriptional regulation of hierarchical vascular tree formation. (**A**) In sprouting angiogenesis, activated endothelial cells upregulate TAL1 to form the transcription factor complex consisting of LMO2 and TAL1 in the nucleus, which upregulates angiopoietin 2, VEGFR2, and DLL4. After detachment of mural cells in response to angiopoietin 2, these cells then preferentially occupy the tip position in vessel sprouts (indicated by red cells). DLL4 on tip cells binds to Notch on adjacent cells to downregulate VEGFR2 (indicated by the dashed line). In contrast, endothelial cells in which LMO2 chiefly binds to LYL1 in the nucleus to transcriptionally upregulate VEGFR1 tend to occupy the stalk position (indicated by blue cells). LYL1 overexpression in tip cells downregulates DLL4 expression and upregulates VEGFR1, which results in hyperbranching of capillaries and slowing of tip cell migration at the periphery of the vascular tree. Only LMO2 partners, TAL1 and LYL1, are described in this figure. (**B**) The oligomeric transcription factor complex composed of E2A, TAL1 or LYL1, LMO2, LDB1 or LDB2, and GATA2 which regulates the expression of a number of downstream angiogenesis-specific genes.

In sprouting angiogenesis, vascular tree formation is a stepwise process of tip cell migration and vessel branching^50^, which is chiefly regulated by the branching pattern generator consisting of VEGFR2-DLL4-Notch^39^. Endothelial cells, especially tip cells in the sprouts, also have an intrinsic transcriptional regulator that may regulate the transition from quiescent to angiogenic endothelium. In quiescent endothelium, which expresses both LMO2 and LYL1 but not TAL1, LMO2 is thought to primarily bind to LYL1 to form the LYL1-LMO2 complex. When LMO2 binds to LYL1, the downstream target angiopoietin 2 is only weakly activated in the quiescent endothelium. In the activated endothelium, which begins to express TAL1, the LMO2 binding partner shifts from LYL1 to TAL1 to form the classical TAL1-LMO2 or TAL1-LMO2-GATA2 transcription factor complexes that initiate vessel sprouting (Fig. 8). After TAL1 is activated in the angiogenic endothelium, the classical LMO2-TAL1 complex becomes a stronger activator of two tip cell proteins, angiopoietin 2 and DLL4, and at the same time suppresses expression of the VEGF-A scavenging receptor, VEGFR1. Moreover, the LMO2-TAL1 complex also upregulates VEGFR2 to augment endothelial cell migration induced by VEGF-A, which is necessary to form relatively straight vessels. Overexpression of the LYL1 protein in tip cells leads to slowing of endothelial cell migration, firstly by upregulating VEGFR1, and secondly by downregulating DLL4 in tip cells, which results in increased branching at the peripheries of the vascular tree (Fig. 8).

As mentioned above, both TAL1-knockout and LMO2-knockout mice exhibited a bloodless phenotype because of the absence of hematopoietic stem cells (HSCs) that normally emerge from the hemogenic endothelium of the dorsal aorta^51^. TAL1 expression becomes dispensable once HSCs are generated^52^. We hypothesize that DLL4 expression on the surface of the hemogenic endothelium of the dorsal aorta stimulates Notch signaling in adjacent cells, future HSCs in this case, which activate the Notch downstream target molecule RUNX1^53,54^.

## Supplementary Information

Supplementary Figure 1.

Supplementary Figure 2.

Supplementary Figure 3.

Supplementary Figure 4.

Supplementary Table 1.

## Data Availability

All data generated or analyzed during this study are included in this published article. All materials are available from the corresponding author on request.
