# Supplementary material for: The transcription factor complex LMO2/TAL1 regulates branching and endothelial cell migration in sprouting angiogenesis
Source: Sci Rep. 2022 May 4;12:7226. doi: 10.1038/s41598-022-11297-3 (PMC9068620; doi:10.1038/s41598-022-11297-3)
Supplement: Supplementary file 1 — Supplementary Figure 1. [file 41598_2022_11297_MOESM1_ESM.pdf]

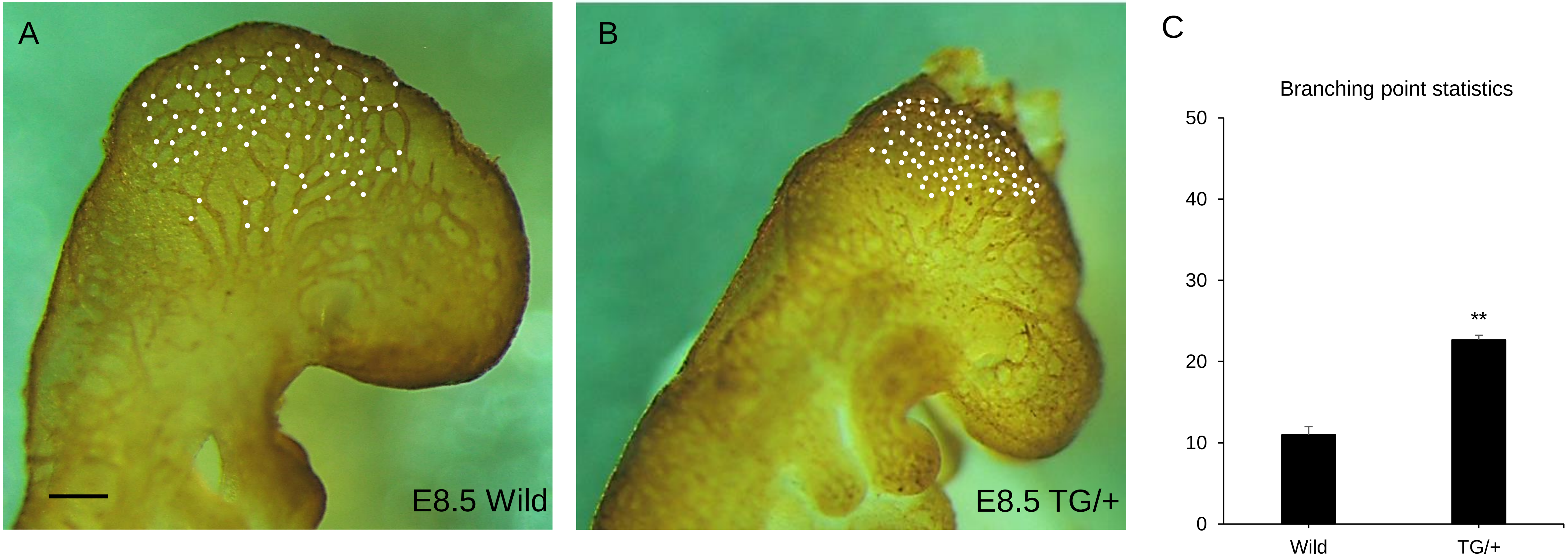

Supplemental Figure 1. Bifurcation points analysis of E8.5 mouse embryo brain blood vessels

TG/+ blood vessels (B) showed more bifurcation (white dots) (**$P < 0.01$, n = 3) in the peripheral part of E8.5 brain than those of wild type littermate (A). C. Statistical analysis of bifurcation points. The vertical axis represents the number of bifurcation per arbitrary field. Bar = 50 μm
