# Supplementary material for: The transcription factor complex LMO2/TAL1 regulates branching and endothelial cell migration in sprouting angiogenesis
Source: Sci Rep. 2022 May 4;12:7226. doi: 10.1038/s41598-022-11297-3 (PMC9068620; doi:10.1038/s41598-022-11297-3)
Supplement: Supplementary file 2 — Supplementary Figure 2. [file 41598_2022_11297_MOESM2_ESM.pdf]

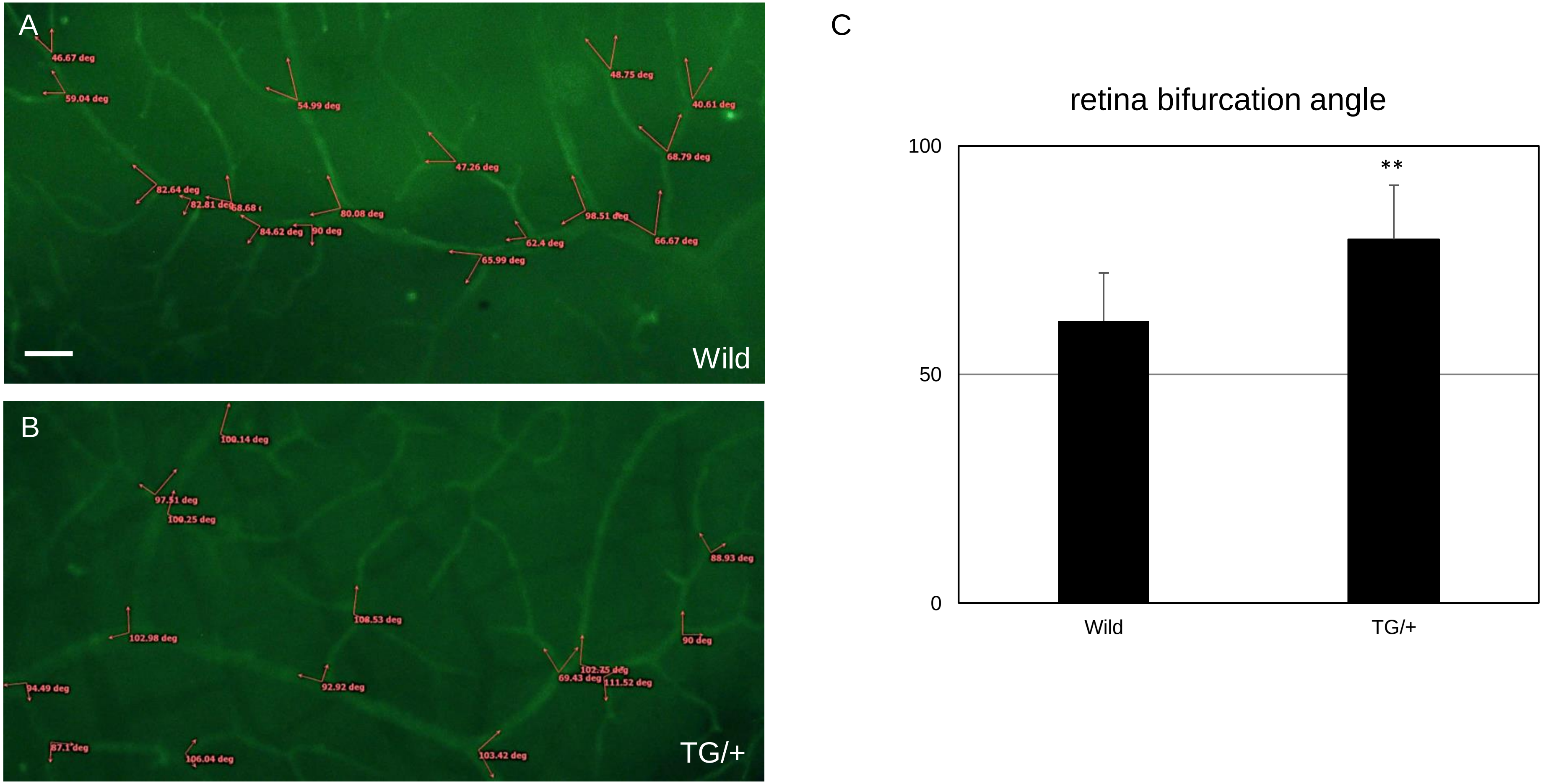

Supplemental Figure 2. Bifurcation angle analysis of 11-week-old mouse retina blood vessel

TG/+ blood vessels (B) showed wider branching angles typical of those at the 11-week-old mouse retina stained with Alexa-Flour 488 Isolectin IB4 compared with those from wild type littermates (A) (**$P < 0.01$, wild n = 66, TG/+ n =55). C. Statistical analysis of bifurcation angle. The vertical axis represents the angle (degrees). Bar = 50 μm
