# Supplementary material for: The transcription factor complex LMO2/TAL1 regulates branching and endothelial cell migration in sprouting angiogenesis
Source: Sci Rep. 2022 May 4;12:7226. doi: 10.1038/s41598-022-11297-3 (PMC9068620; doi:10.1038/s41598-022-11297-3)
Supplement: Supplementary file 3 — Supplementary Figure 3. [file 41598_2022_11297_MOESM3_ESM.pdf]

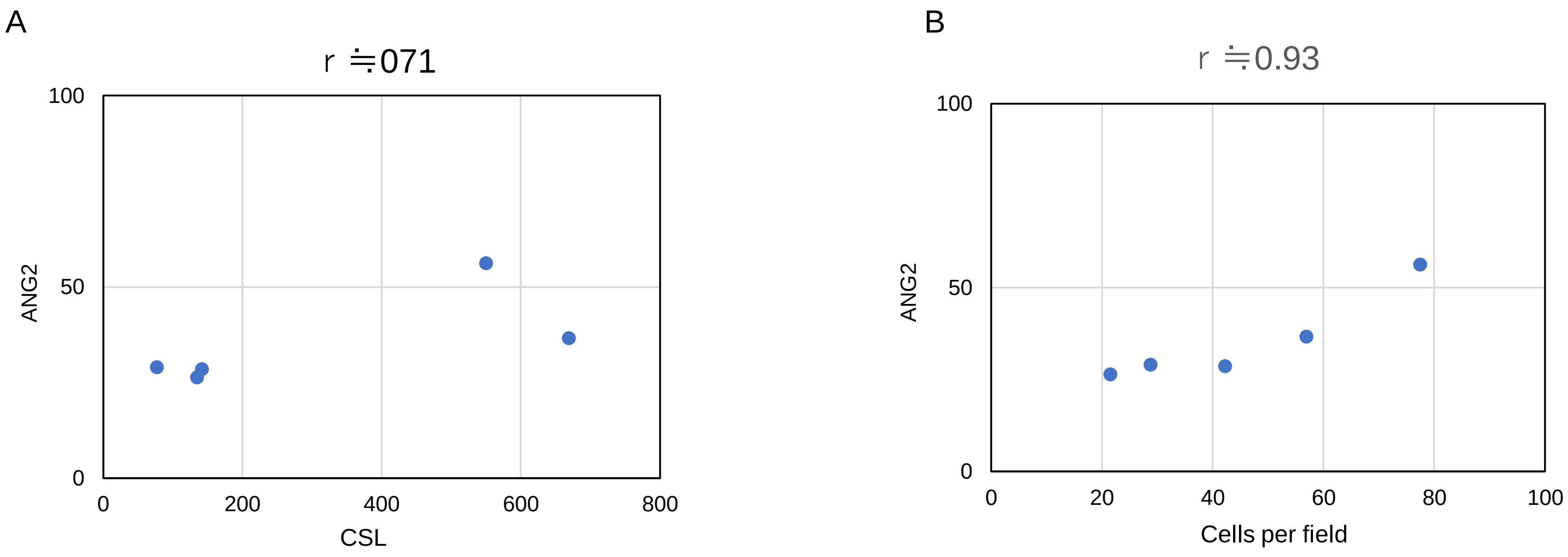

Supplemental Figure 3. Correlation between secreted angiopoietin 2 level and endothelial migration

Angiopoietin 2 levels in the supernatant showed a strong correlation with CSL in spheroids treated with each siRNA (correlation coefficient: r = 0.71) (A) and number of cells per field in endothelial cell invasion assay of HUVEC treated with each siRNA (r = 0.92)(B).
