# Supplementary material for: The transcription factor complex LMO2/TAL1 regulates branching and endothelial cell migration in sprouting angiogenesis
Source: Sci Rep. 2022 May 4;12:7226. doi: 10.1038/s41598-022-11297-3 (PMC9068620; doi:10.1038/s41598-022-11297-3)
Supplement: Supplementary file 4 — Supplementary Figure 4. [file 41598_2022_11297_MOESM4_ESM.pdf]

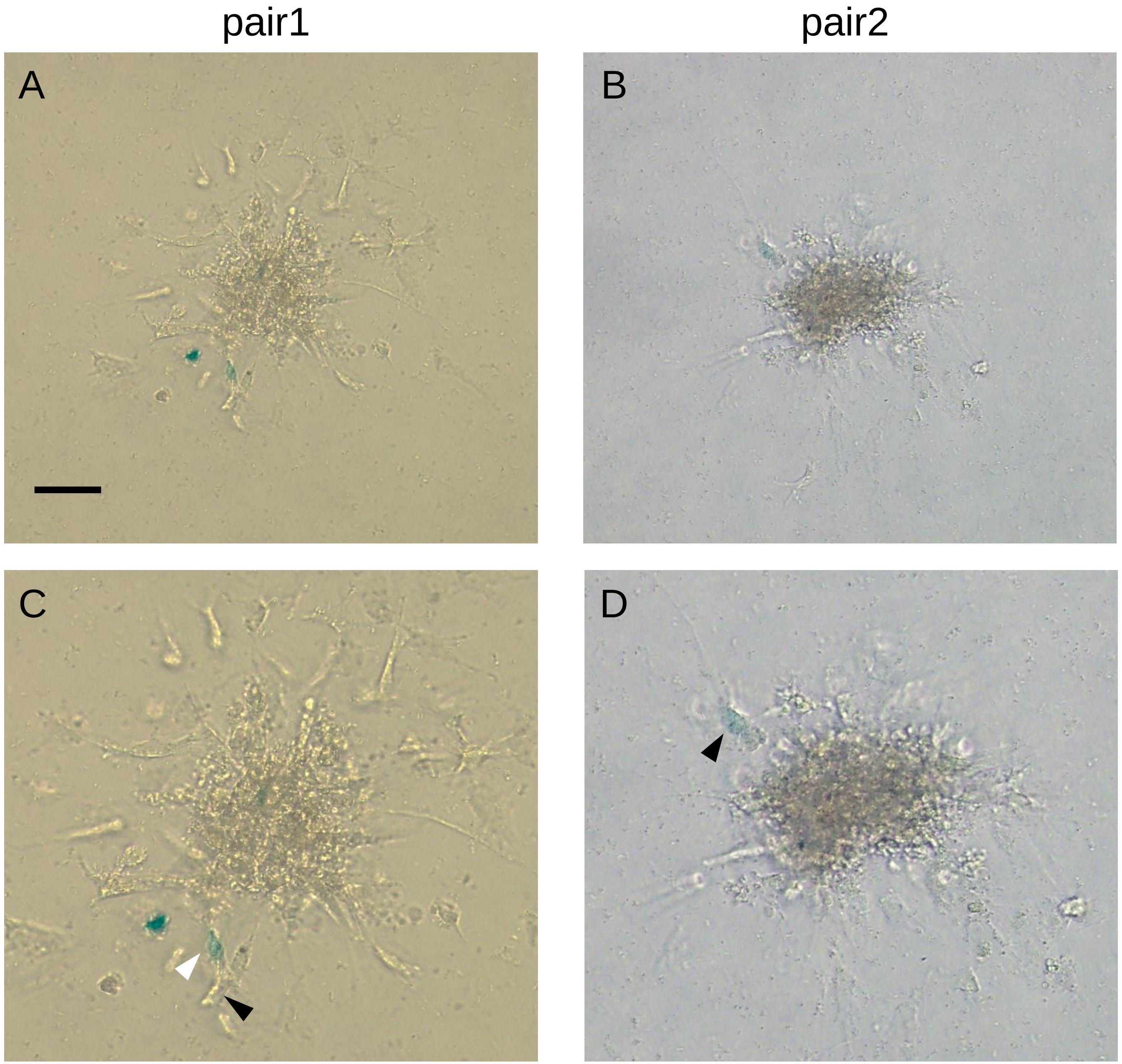


**Supplemental Figure 4. LYL1-expressing cells preferentially occupy the stalk position over TAL1-expressing cells in vessel sprouts from HDMECs spheroids *in vitro*.**

Cultured HDMECs were transfected with a mammalian expression vector encoding LYL1, TAL1, and β-galactosidase. In pair1, non-labeled TAL1-expressing cells were mixed at 1:1 ratio with β-galactosidase-labeled LYL1-expressing cells. In pair2, β-galactosidase-labeled TAL1-expressing cells were mixed at 1:1 ratio with non-labeled LYL1-expressing cells. Before spheroids formation pair1 and pair2 HDMECs were mixed with non-labeled HUVECs at 4:1 ratio. Both chimeric spheroids were cultured in type 1 collagen gel for 24 hours with 20 ng/ml VEGF-A stimulation. Labeled cells were detected by whole mount X-gal staining immediately after culture. Cells at the tip position in the sprouts are indicated by black arrowheads and those at the stalk position by white arrowheads. A., C. Representative images of X-gal-stained pair1 spheroids. The labeled cells (LYL1-expressing cells in this pair) were preferentially located in the stalk position. B., D. Representative images of X-gal-stained pair2 spheroids. The labeled cells (TAL1-expressing cells) tend to occupy the tip position. The distributions of labeled cells in all chimeric spheroids are shown in Table 2. Relatively weak X-gal staining was due to slight degeneration of HDMECs during sprouting. Bar = 100 μm.
