# Supplementary material for: The transcription factor complex LMO2/TAL1 regulates branching and endothelial cell migration in sprouting angiogenesis
Source: Sci Rep. 2022 May 4;12:7226. doi: 10.1038/s41598-022-11297-3 (PMC9068620; doi:10.1038/s41598-022-11297-3)
Supplement: Supplementary file 5 — Supplementary Table 1. [file 41598_2022_11297_MOESM5_ESM.pdf]

Supplemental table

Primers for real-time qPCR

	Forward	Reverse
GAPDH	ACACCCACTCCTCCACCTTT	TCCACCACCCTGTTGCTGTA
LMO2	TACTTCCTGAAGGCCATC	CCTGAGATAGTCTCTCCG
TAL1	CTCGGCAGCGGGTTCTTTGG	CTCCATCTCATAGGGGGAAG
LYL1	CATCTTCCCTAGCAGCCGGTTG	GTTGGTGAACACGCGCCG
GATA2	ATCCAGACTCGGAACCGGAAG	GCACTTTGACAGCTCCTCGAAG
VEcadh	CGCCTCTGTCATGTACCAAA	GTCTTCAGGCACGACAAATG
NRP2	CTGTGGGTCATCCGTGAGGAC	ATGGGTTCCATGCAGTTCTCCAG
NRP1	ACACCTGAGCTGCGGACTTT	GGCCTGGTCGTCATCACAT
VEGFR1	CTGTCATGCTAATGGTGTCCC	TGCTGCTTCCTGGTCCTAAAATA
VEGFR2	CACCACTCAAACGCTGACATGTA	CCAACTGCCAATACCAGTGGA
VEGFR3	CAAGGCCAACAACGGCAT	TCGACGCTGATGAAGGGA
HPRT1	GGTGAAAAGGACCCCACGAA	GTATTCATTATAGTCAAGGGC
